# The dynamic change of tuberculosis infection prevalence in rural residents: 10-year follow-up of a population-based, multicentre cohort study from China

**DOI:** 10.1016/j.lanwpc.2025.101509

**Published:** 2025-03-06

**Authors:** Xuefang Cao, Lei Gao, Henan Xin, Limei Zhu, Weitao Duan, Boxuan Feng, Wei Lu, Zisen Liu, Yijun He, Lingyu Shen, Juanjuan Huang, Bin Zhang, Dakuan Wang, Jiaoxia Yan, Cheng Chen, Lihui Wang, Wenhua Yin, Guochen Wang, Tonglei Guo, Yuanzhi Di, Zihan Li, Jianguo Liang, Yaqi Zhao, Hongzhi Li, Fei Shen, Jiang Du, Qi Jin

**Affiliations:** a NHC Key Laboratory of Systems Biology of Pathogens, National Institute of Pathogen Biology, and Center for Tuberculosis Research, Chinese Academy of Medical Sciences and Peking Union Medical College, Beijing, P.R. China; b Key Laboratory of Pathogen Infection Prevention and Control (Ministry of Education), National Institute of Pathogen Biology, Chinese Academy of Medical Sciences & Peking Union Medical College, Beijing, P.R. China; c Center for Disease Control and Prevention of Jiangsu Province, Nanjing, P.R. China; d Center for Disease Control and Prevention of Zhongmu County, Zhengzhou, P.R. China; e Center for Disease Control and Prevention of Danyang City, Danyang, P.R. China; f The Sixth People's Hospital of Zhengzhou, Zhengzhou, P.R. China

**Keywords:** Tuberculosis infection, 10-year follow-up, Multicentre cohort study, China

## Abstract

**Background:**

The incidence of tuberculosis (TB) decreased significantly in recent years in China. However, the declining in the burden of tuberculosis infection (TBI) have not been systematically evaluated. The aim of this study was to elucidate the changes of TBI prevalence during the past decade.

**Methods:**

Based on a population-based, multicenter cohort study (LATENTTB-NSTM), a 10-year follow-up survey was conducted among registered residents (≥18 years old) at two study sites (Zhongmu and Danyang) using open-cohort design. After excluding active TB, tuberculin skin test (TST) and interferon-γ release assay (IGRA) were used to determine TBI status of each participant.

**Findings:**

Overall, 5924 eligible participants who completed the follow-up survey were included in the analysis. Compared to the age- and gender-standardized TBI prevalence determined by IGRA in 2013, the prevalence of TBI was observed to be decreased by 22·24% (from 15·11% to 11·75%) in Danyang site and by 40·86% (from 16·57% to 9·80%) in Zhongmu site in 2023. A consistently declining trend was observed as well for TBI prevalence determined by TST test. The acquisition of TBI in 10 years was assessed by the conversion rate of IGRA result in 4648 participants who participated in both the 2013 and 2023 surveys. The IGRA conversion rate in Danyang site was significantly higher than that in Zhongmu site (4% vs. 2%, p < 0·0001). The reversion rate of IGRA result was assessed as well, and no statistically significant difference was observed between the two study sites (29% in Danyang site vs. 31% in Zhongmu site, p = 0·577). Male gender was found to be associated with an increased risk of IGRA conversion as compared to female, with adjusted odds ratio (OR) of 1·46 (95% confidence interval [CI]: 1·00–2·13). In addition, never smokers were observed to be associated with significantly higher IGRA reversion rates (OR = 2·91, 95% CI: 1·52–5·57) (p = 0.001) as compared to current smokers. We also found the influence of BCG vaccination at birth on TST positivity was non-significant among individuals aged 15 and above.

**Interpretation:**

Our findings suggest that the prevalence of TBI in rural residents from China has significantly decreased along with the declining of TB incidence in the last decade. The downward trend shows regional differences, which might be partly explained by the difference in new infection rates across regions.

**Funding:**

The CAMS Innovation Fund for Medical Sciences and the 10.13039/501100001809National Natural Science Foundation of China.

**Research in context**

**Evidence before this study**

Over the past decade, China has adopted a series of prevention and control measures to reduce the incidence of tuberculosis (TB). With the implementation of these measures, the incidence rate of TB has decreased by 37% since 2013 (from 69·42 per 100,000 in 2013 to 43·49 per 100,000 in 2023) in China. It is worth exploring how the burden of tuberculosis infection (TBI) changes, which is crucial for making more timely and precise TB prevention and control strategies.

We searched PubMed for articles published in English up to Feb 7, 2025, with “latent tuberculosis infection” OR “latent” AND “tuberculosis” and “China” as search terms. Of the 879 articles we identified, 126 were meta-analyses or reviews, and 699 did not epidemiologically investigate TBI in mainland China. Of the 54 eligible articles, only one population-based multicentre cohort study was conducted to evaluate the prevalence of TBI in rural China (the baseline data of this study).

**Added value of this study**

In this study, we observed that with the optimization of the TB control strategy in China, there has been a significant decline in both the incidence of TB and TBI prevalence in rural population. Importantly, the downward trend showed regional variations, which might be partly due to differences in the rates of new infections across regions.

**Implications of all the available evidence**

Our data suggest that the use of comprehensive TB prevention and control strategies, including proactive TB case finding and promotion of TB preventive treatment, can be expected to contribute to accelerating achievement of the WHO End TB Strategy goal of reducing the incidence of TB to less than 10 per 100,000 by 2035.

## Introduction

Tuberculosis (TB) remains one of the world's top infectious killers. In 2023, about 741,000 people fell ill with TB in China, the third highest number of cases in the world.^1^ It is estimated that around a quarter of the world's population is infected with *Mycobacterium tuberculosis*.^2^ Following acquisition of infection, 5%–10% of people with tuberculosis infection (TBI) might develop active disease.^3^^,^^4^ Management of TBI in subgroups at high-risk of developing active disease is therefore an emerging priority intervention to promote the achievement of the End TB goals. As a country with high-burden of TB, China needs to clarify the burden and the epidemic characteristics of TBI, which is the premise for developing appropriate national strategies and guidelines on TBI management. Therefore, we launched a population-based, multicentre, prospective cohort study (LATENTTB-NSTM) in registered rural residents (≥5 years old) at four study sites in 2013. The study found that between the study sites, the age- and gender-adjusted positivity rates of tuberculin skin test (TST) (≥10 mm) ranged from 15% to 42%, and the age- and gender-adjusted positivity rates of interferon-γ release assays (IGRA) ranged from 13% to 20%.^5^ Stratified analysis consistently showed that the national Bacille Calmette-Guérin (BCG) vaccination programme significantly influenced the positivity of the TST, especially in participants with booster vaccination.^6^

Over the past decade, China has adopted a series of prevention and control measures to decrease the incidence of TB. For example, in 2019, the Action Plan to Stop Tuberculosis (2019–2022) was formulated in China.^7^ In order to implement this policy, in addition to close contacts of microbiologically confirmed TB patients and people living with HIV/AIDS, active screening for TB has been launched in many regions for the elderly (aged ≥65 years) and diabetics. In 2020, the Guideline for the Prevention and Control of Tuberculosis in China's Schools was released, and TB-related tests have been included in physical examinations for new students and school staff.^8^ With such continuous improvement in TB prevention and control policies, the incidence rate of TB has decreased 37% since 2013 (from 69·42 per 100,000 in 2013 to 43·49 per 100,000 in 2023) in China.^9^ In addition, the demographic characteristics of China have changed as well, with the proportion of elderly people increasing. In this context, it is highly necessary to investigate the changes that have occurred in the burden of TBI over the past decade. Therefore, the aim of this study was to elucidate the changes in the prevalence of TBI based on the 10-year follow-up survey of the LATENTTB-NSTM study, which might contribute to a more comprehensive understanding of the dynamic changes in the TB epidemic.

## Methods

### Study design and participants

Based on a population-based, multicenter cohort study (LATENTTB-NSTM) launched in 2013, the 10-year follow-up survey using open-cohort design was conducted among registered residents at two study sites (Danyang, Jiangsu Province and Zhongmu, Henan Province) between 1 October 2023 and 31 January 2024 to screen for TBI using both TST and IGRA (QuantiFERON Gold In-Tube [QFT, Qiagen, Hilden, Germany]).^5^ All eligible current residents living in the two selected study sites were included in 10-year follow-up survey to assess the prevalence of TBI in 2023. In addition, the subset of the study population, who completed the testing in both the baseline survey (2013) and the 10-year follow-up survey (2023), was used to estimate the rates of QFT conversion and reversion. Inclusion criteria of the study participants for analysis of TBI included: birth before 1 June 2005 (≥18 years old), household registration or residence permit, continuous residence at the study site for 6 months or more in the previous year, ability to complete the survey and tests during the study period, and voluntary signing of the informed consent form. Exclusion criteria: current active TB, history of TB and pregnancy.

We also tracked the changes in TST results for participants aged 5–14 years in 2013 and also tested the TST for participants aged 5–24 years in 2023, with the primary aim of assessing how long the effect of BCG vaccination on TST results.

The study protocol was approved by the ethics committees of the Institute of Pathogen Biology, Chinese Academy of Medical Sciences, Beijing, China (approval IPB-2023-35).

### Procedure

In this study, measures were taken to ensure data quality and comparability between the two study sites, including standardization of research protocols, uniform training of researchers, using of reagents with the same batch number and in standardized experimental procedures.

For each study participant, socio-demographic information was collected using a standardized questionnaire administered by trained interviewers, including age, gender, level of education, smoking status, alcohol consumption status, weight, height and presence of a BCG scar. Self-reported history of TB disease, self-reported history of close contact with TB patients, self-reported history of immunological diseases and history of pulmonary diseases (including chronic obstructive pulmonary disease, emphysema or chronic bronchitis) were also collected. Present TB and previous TB history were verified based on the national active TB case report system.

TBI was tested by QFT and TST for each participant. Venous blood was collected in lithium heparin tubes, QFT test was performed strictly according to the manufacturer's recommendations, with a cut-off value of 0·35 IU/mL or more.

TST was performed after blood collection using the Mantoux method—ie, injection of 0·1 mL of 5 tuberculin units of purified protein derivative (PPD) (Xiangrui; Beijing, China) was preferentially injected intradermally into the left forearm.^10^ Trained study personnel measured the size of the tuberculin reaction (induration) in mm after 48–72 h of placement, with indentation diameters ≥10 mm being considered positive. Participants with skin diseases on their forearms did not undergo TST.

Chest X-ray examination was performed and suspected symptoms of pulmonary TB were investigated on each enrolled participant to identify suspected patients with active pulmonary TB. All suspected TB cases were transferred to the local Center for Disease Control and Prevention for confirmation. Participants with suspected pulmonary infection (defined by radiographic abnormalities consistent with active pulmonary infection together with a positive QFT test or a strong positive TST [induration diameter ≥15 mm]) were not included in the analysis of TBI.^5^

### Statistical analysis

The data were entered by two independent professional data-entry workers using EpiData software (EpiData Version 3.1, EpiData Association Odense, Denmark). For any inconsistencies between the two databases, the original data would be checked for confirmation. Statistical analyses were performed using the Statistical Analysis System (SAS 9.4; SAS Institute Inc., NC, USA) and GraphPad Prism 8 (GraphPad Software, San Diego, CA).

Quantitative data are expressed as median values and interquartile ranges (IQR), and qualitative data are expressed as numbers (percentages). Body mass index (BMI) was calculated as weight for height squared (kg/m^2^). The traditional definition of IGRA reversion was that the level of released IFN-γ in IGRA decreased from ≥0·35 IU/mL in 2013 to <0·35 IU/mL in 2023. The stricter definition of IGRA reversion was that the level of released IFN-γ in IGRA decreased from >0·70 IU/mL in 2013 to <0·20 IU/mL in 2023. The traditional definition of IGRA conversion was that the level of released IFN-γ in IGRA increased from <0·35 IU/mL in 2013 to ≥0·35 IU/mL in 2023. The stricter definition of IGRA conversion was that the level of released IFN-γ in IGRA increased from <0·20 IU/mL in 2013 to >0·70 IU/mL in 2023.^11^ Data on the incidence of active TB at both sites between 2013 and 2023 were obtained from the national active TB case report system.

Fisher's exact test and Pearson's chi-squared (χ^2^) test were used to compare the distribution of categorical variables between groups. Age and gender were fixed in multivariable models, and other variables showing significant associations (p < 0.20) in univariable analysis were included in the unconditional multiple logistic regression analyses, with associations being presented as odds ratio (OR) and 95% confidence interval (CI). The GM (1,1) model is a time series prediction model that can accurately predict monotonic processes,^12^ which was used to estimate and predict the incidence of active TB in this study. The agreement between QFT and TST was evaluated using Cohen's kappa coefficient and 95% CI.

## Results

### Characteristics of the study participants included in the analysis of TBI in 2023

The information on the two study sites and the study participants included in the analysis of TBI were showed in detail in Appendix p 2. Of the 7364 eligible participants, 6031 actually participated in the 10-year follow-up survey, giving a response rate of 82% (Appendix p 2). After exclusion of 107 participants with a history of TB, clinically suspected pulmonary TB or incomplete data, 5924 participants were included in the final analysis (Appendix p 2). About half of the participants were female gender and age ≥60 years (Table 1). The age distribution was significantly different between the two study sites (p < 0·0001), with a higher proportion of people aged ≥60 years in Danyang than in Zhongmu (Table 1). 24% (1424/5924) of participants reported current smoking and 24% (1400/5924) had consumed alcohol in the past year (Table 1).

**Table 1:** Characteristics of the study participants included in the TBI detection in 2023.

Variables^a^	Total (N = 5924)	Danyang site (N = 3094)	Zhongmu site (N = 2830)	p for χ^2^ test^c^
**Age**				<0·0001
18–29 years	232 (4%)	44 (1%)	188 (7%)	
30–39 years	490 (8%)	79 (3%)	411 (14%)	
40–49 years	563 (10%)	203 (7%)	360 (13%)	
50–59 years	1680 (28%)	827 (27%)	853 (30%)	
60–69 years	1554 (26%)	973 (31%)	581 (21%)	
≥70 years	1405 (24%)	968 (31%)	437 (15%)	
**Gender**				0·440
Female	3081 (52%)	1624 (52%)	1457 (51%)	
Male	2843 (48%)	1470 (48%)	1373 (49%)	
**Highest education level**				<0·0001
Primary school or lower	2405 (40%)	1218 (40%)	1187 (42%)	
Middle school	2536 (43%)	1419 (46%)	1117 (40%)	
High school	705 (12%)	384 (12%)	321 (11%)	
College or higher	278 (5%)	73 (2%)	205 (7%)	
**BMI**				<0·0001
<18·5 kg/m^2^	87 (2%)	69 (2%)	18 (1%)	
18·5–<24 kg/m^2^	2023 (34%)	1264 (41%)	759 (27%)	
24–<28 kg/m^2^	2441 (41%)	1298 (42%)	1143 (40%)	
≥28 kg/m^2^	1373 (23%)	463 (15%)	910 (32%)	
**Smoking**				0·003
Never smoker	4018 (68%)	2112 (68%)	1906 (67%)	
Former smoker	482 (8%)	217 (7%)	265 (10%)	
Current smoker	1424 (24%)	765 (25%)	659 (23%)	
**Alcohol drinking**				<0·0001
No	4524 (76%)	2256 (73%)	2268 (80%)	
Yes	1400 (24%)	838 (27%)	562 (20%)	
**Number of BCG scars**				<0·0001
0	2625 (45%)	2239 (72%)	386 (14%)	
1	1799 (30%)	678 (22%)	1121 (40%)	
≥2	1463 (25%)	177 (6%)	1286 (46%)	
**History of pulmonary diseases**^b^				<0·0001
No	5766 (97%)	2978 (96%)	2788 (99%)	
Yes	158 (3%)	116 (4%)	42 (1%)	
**Self-reported history of close contact with patient with TB**				0·068
No	5900 (99%)	3077 (99%)	2823 (99%)	
Yes	24 (1%)	17 (1%)	7 (1%)	
**Self-reported history of immunological diseases**				<0·0001
No	5851 (99%)	3033 (98%)	2818 (99%)	
Yes	73 (1%)	61 (2%)	12 (1%)	
**QFT test**				<0·0001
Negative	5079 (86%)	2577 (83%)	2502 (88%)	
Positive	793 (13%)	476 (16%)	317 (11%)	
Indeterminate	52 (1%)	41 (1%)	11 (1%)	
**TST induration**				<0·0001
<5 mm	4544 (80%)	2346 (80%)	2198 (79%)	
5–9 mm	255 (4%)	97 (3%)	158 (6%)	
10–14 mm	337 (6%)	207 (7%)	130 (5%)	
≥15 mm	569 (10%)	278 (10%)	291 (10%)	

Abbreviations: TBI, tuberculosis infection; BMI, body mass index; BCG, bacille Calmette-Guerin; TB, tuberculosis; QFT, QuantiFERON-TB Gold In-Tube; TST, tuberculin skin test.

a Data might not sum to total because of missing data. The missing frequency of BCG scars and TST were 0.62% (37/5924) and 3.70% (219/5924), respectively. The missing data were not included in the χ^2^ test between the two sites.

b Including chronic obstructive pulmonary disease, emphysema or chronic bronchitis.

c A two-tailed p value < 0.05 was considered statistically significant.

### Changes in TBI prevalence and TB incidence over the past 10 years

4% (219/5924) of participants did not complete the TST test due to missing measurements or refusal to inject PPD. Overall, 13% (793/5924) of participants were QFT positive and 16% (906/5705) of participants were TST positive (induration diameter ≥10 mm) (Table 1). Agreement between TST and QFT was poor, with 5634 participants with valid results showing a kappa coefficient of 0·35 (95% CI: 0·31, 0·38) (Appendix p 3). Increasing age and current smoking were associated with increased risk of QFT positivity, while having two or more BCG scars (compared with no BCG scars) was associated with decreased risk of QFT positivity (Appendix p 4).

**Fig. 1:**
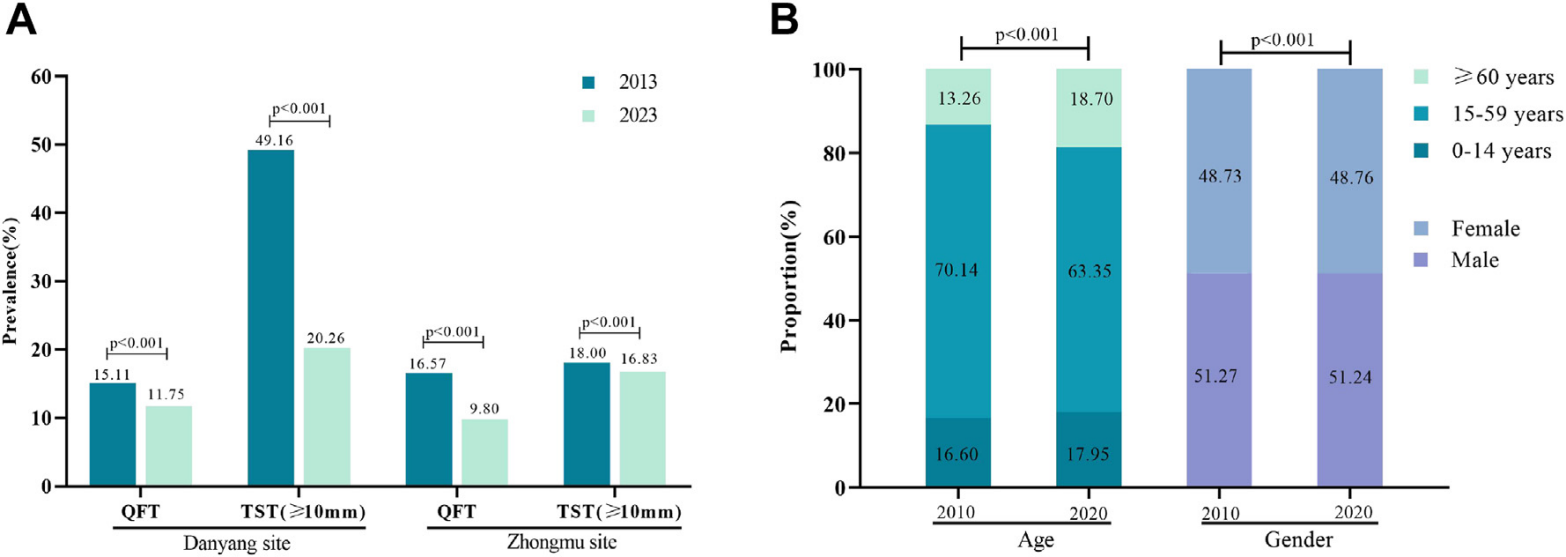
**Age- and gender-standardised rate of QFT positivity and TST positivity (≥10 mm) based on national census data as standard population**. (A) Comparison of the rates of QFT positivity or TST positivity between 2013 and 2023, standardised by age and gender based on national census data. Bars indicate age- and gender-standardised positivity rate of QFT or TST. (B) Comparison of 2010 and 2020 national census data by age and gender, respectively. Bar charts show the proportion of different age groups or genders.

As for changes in TBI prevalence, after standardisation for age and gender using 2010 and 2020 national census data, respectively, the TBI prevalence detected by QFT has decreased from 15·11% to 11·75% in Danyang site, with an annual decrease of 2.48% between 2013 and 2023 (Fig. 1; Appendix p 5). The age- and gender-standardised TST positivity rates has decreased from 49·16% to 20·26% in Danyang site (Fig. 1). After standardisation for age and gender, TBI prevalence detected by QFT has decreased from 16·57% to 9·80% in Zhongmu site, with an annual decrease of 5.12% between 2013 and 2023 (Fig. 1; Appendix p 5). The age- and gender-standardised TST positivity rates has decreased from 18·00% to 16·83% in Zhongmu site (Fig. 1).

**Fig. 2:**
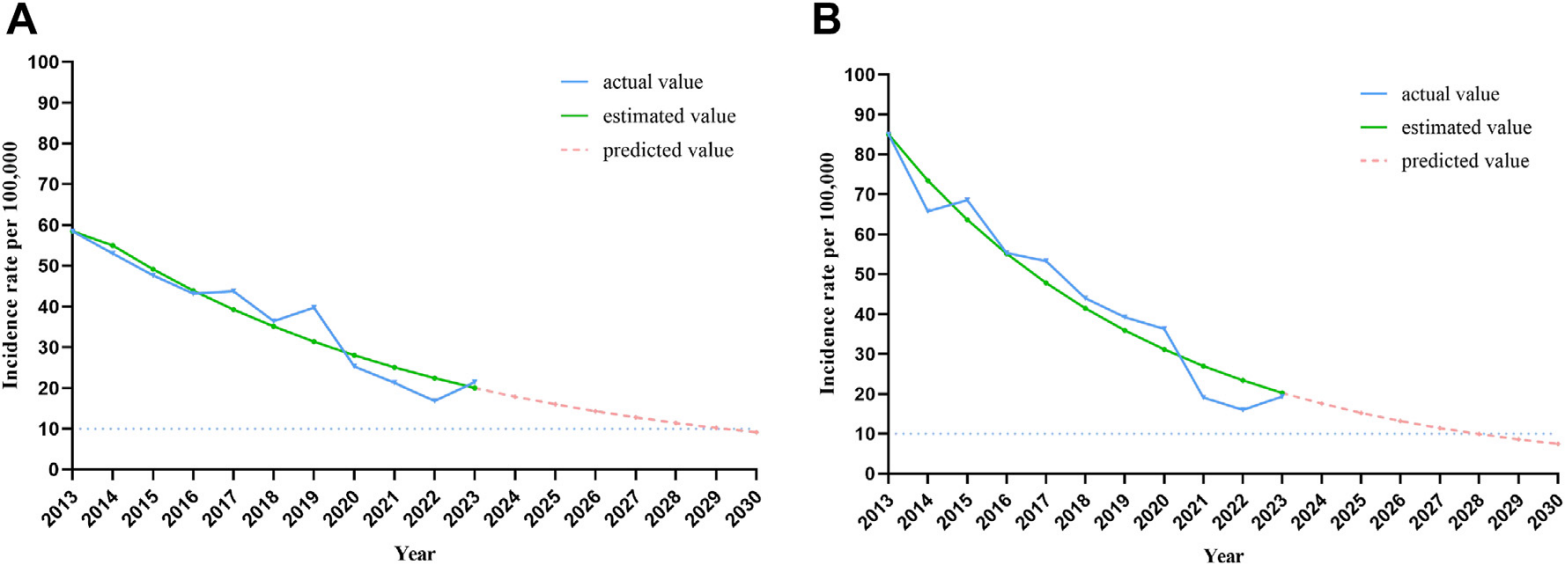
**The change of reported TB incidence rates for the Danyang and Zhongmu sites between 2013 and 2030**. Based on the reported TB incidence rates in Danyang site (A) and Zhongmu site (B) from 2013 to 2023 (actual values), the GM (1,1) model was used to estimate the TB incidence rates from 2013 to 2023 (estimated values) and to predict the trend in TB incidence rates over the next 7 years (predicted values). The blue solid line represents the actual values. The green solid line represents the estimated values, and the red dotted line represents the predicted values.

As for changes in TB incidence, the reported incidence of active TB has fallen by 63·35% between 2013 and 2023 in Danyang site, with an annual rate of decline being 9·55% (Appendix p 5). The reported incidence of active TB has fallen by 77·24% between 2013 and 2023 in Zhongmu site, with an annual rate of decline being 13·76% (appendix p 5). The results of the GM (1,1) model were shown in Fig. 2, which showed that the incidence of active TB in both sites could fall below 10/100,000 in 2030.

### QFT conversion at 10-year follow-up among participants who participated in the 2013 and 2023 examinations

A total of 4648 people participated in the 2013 and 2023 surveys (Appendix p 6). As shown in Table 2, 3% (112/3717) of participants with negative QFT results in 2013 were found to be positive in 2023. The QFT conversion rate at 10-year follow-up was lower in Zhongmu site (2%) than in Danyang site (4%) (OR = 0·39, 95% CI: 0·23–0·66). A significantly higher incidence of QFT conversion was found in males (OR = 1·46, 95% CI: 1·00–2·13) (Table 2). The same result was obtained using a stricter definition of conversion, but it was not statistically significant (OR = 1·61, 95% CI: 0·88–2·94) (Appendix p 8). 0·36% (11/3023) of participants with baseline QFT-/TST-showed positive results for both QFT and TST in 2023 (Appendix p 9).

**Table 2:** Identification of potential factors associated with conversion of QFT by traditional definition among participants with 2013 and 2023 examinations.

Variables^a^	QFT conversion rate^b^, n/N (%)	p value^c^	Adjusted OR^f^ (95% CI)
**Total**	112/3717 (3%)		
**Age**		0·398^d^	
<60 years	53/1905 (3%)		Reference
≥60 years	59/1812 (3%)		0·80 (0·53–1·21)
**Gender**		0·112^d^	
Female	52/2000 (3%)		Reference
Male	60/1717 (4%)		1·46 (1·00–2·13)
**Highest education level**		0·287^d^	
High school or lower	105/3559 (3%)		
College or higher	7/158 (4%)		
**BMI**		0·311^d^	
<18·5 kg/m^2^	2/55 (4%)		
18·5–<24 kg/m^2^	34/1249 (3%)		
24–<28 kg/m^2^	55/1524 (4%)		
≥28 kg/m^2^	21/889 (2%)		
**Smoking**		0·728^d^	
Never smoker	74/2554 (3%)		
Former smoker	9/316 (3%)		
Current smoker	29/847 (3%)		
**Alcohol drinking**		0·658^d^	
No	89/2890 (3%)		
Yes	23/827 (3%)		
**Self-reported history of close contact with patient with TB**		1·000^e^	
No	112/3699 (3%)		
Yes	0/18 (0%)		
**Self-reported history of immunological diseases**		1·000^e^	
No	111/3673 (3%)		
Yes	1/44 (2%)		
**BCG scar**		<0·0001^d^	
Absent	72/1680 (4%)		Reference
Present	39/2036 (2%)		0·68 (0·41–1·13)
**Study site**		<0·0001^d^	
Danyang	82/1836 (4%)		Reference
Zhongmu	30/1881 (2%)		0·39 (0·23–0·66)

Abbreviations: QFT, QuantiFERON-TB Gold In-Tube; OR, odds ratio; CI: confidence interval; BMI, body mass index; TB, tuberculosis; BCG, bacille Calmette-Guerin.

a Data might not sum to total because of missing data.

b The definition of conversion: the IFN-γ levels of TBAg-Nil from <0·35 IU/mL in 2013 to ≥0·35 IU/mL in 2023.

c A two-tailed p value < 0·20 was considered statistically significant.

d p for χ^2^ test.

e p for Fisher's exact test.

f The missing cases were removed from the multivariable model. Age and gender were fixed in the multivariable models, other variables with p < 0·20 in the univariate model were also entered into the multivariable models.

### QFT reversion at 10-year follow-up among participants who participated in the 2013 and 2023 examinations

Using the traditional definition of QFT reversion, 46% (431/931) of QFT positive subjects in 2013 were found to be negative in 2023. Factors associated with QFT reversion were elderly participants and current smoking (Appendix p 10). Using the stricter definition of QFT reversion, the QFT reversion rates at 10-year follow-up were 29% at Danyang site and 31% at Zhongmu site (p = 0·577). A significantly higher rate of QFT reversion was found for never smokers (OR = 2·91 95% CI: 1·52–5·57) (Table 3). 22·41% (130/580) of participants with baseline QFT+/TST + showed negative results for both QFT and TST in 2023 (Appendix p 9).

**Table 3:** Identification of potential factors associated with reversion of QFT by stricter definition among participants with 2013 and 2023 examinations.

Variables^a^	QFT reversion rate^c^, n/N (%)	p value^d^	Adjusted OR^g^ (95% CI)
**Total**	150/499 (30%)		
**Age**		0·006^e^	
<60 years	40/178 (22%)		Reference
≥60 years	110/321 (34%)		1·69 (1·10–2·61)
**Gender**		0·928^e^	
Female	69/228 (30%)		Reference
Male	81/271 (30%)		1·73 (0·98–3·06)
**Highest education level**		0·446^f^	
High school or lower	149/491 (30%)		
College or higher	1/8 (13%)		
**BMI**		0·141^f^	
<18·5 kg/m^2^	3/4 (75%)		6·71 (0·66–67·83)
18·5–<24 kg/m^2^	58/177 (33%)		Reference
24–<28 kg/m^2^	63/216 (29%)		0·85 (0·54–1·32)
⩾28 kg/m^2^	26/102 (25%)		0·67 (0·38–1·17)
**Smoking**		0·006^e^	
Never smoker	96/294 (33%)		2·91 (1·52–5·57)
Former smoker	26/66 (39%)		2·83 (1·45–5·51)
Current smoker	28/139 (20%)		Reference
**Alcohol drinking**		0·148^e^	
No	118/371 (32%)		Reference
Yes	32/128 (25%)		0·78 (0·46–1·31)
**History of pulmonary diseases**^b^		0·095^f^	
No	144/488 (30%)		Reference
Yes	6/11 (55%)		2·93 (0·86–10·04)
**Self-reported history of immunological diseases**		0·371^f^	
No	147/493 (30%)		
Yes	3/6 (50%)		
**BCG scar**		0·617^e^	
Absent	80/260 (31%)		
Present	66/230 (29%)		
**Study site**		0·577^e^	
Danyang	75/259 (29%)		
Zhongmu	75/240 (31%)		

Abbreviations: QFT, QuantiFERON-TB Gold In-Tube; OR, odds ratio; CI: confidence interval; BMI, body mass index; BCG, bacille Calmette-Guerin.

a Data might not sum to total because of missing data.

b Including chronic obstructive pulmonary disease, emphysema or chronic bronchitis.

c The definition of reversion: the IFN-γ levels of TBAg-Nil from >0·70 IU/mL in 2013 to <0·20 IU/mL in 2023.

d A two-tailed p value < 0·20 was considered statistically significant.

e p for χ^2^ test.

f p for Fisher's exact test.

g The missing cases were removed from the multivariable model. Age and gender were fixed in the multivariable models, other variables with p < 0·20 in the univariate model were also entered into the multivariable models.

Among the people aged ≥18 years who participated in both baseline survey and 10-year follow-up survey, 9 of 526 and 13 of 433 people with TBI developed TB in Danyang and Zhongmu, respectively.

### The effect of BCG vaccination on TST results

We analysed the relationship between TST and BCG vaccination in participants aged 5–14 years in 2013 and 2023, respectively. We found that TST positivity (≥10 mm) was higher among participants with BCG scars compared to those without BCG scars, although this did not reach significant difference (in 2013: 11% vs. 6%, p = 0·348; in 2023: 4% vs. 0%, p = 0·540) (Appendix p 11). However, for participants aged 15–24 years, TST positivity (≥10 mm) was similar between participants with and without BCG scars (Appendix p 11).

## Discussion

Based on a cohort established in 2013, changes of TBI prevalence over the past decade in rural residents from China were investigated, with the open cohort design for assessing of changes in TBI burden and with the closed cohort design for observing of outcomes of infection. In present study, we found that the age- and gender-standardized positive rates of QFT test and TST test were lower in 2023 than that in 2013 at both study sites, with the decreasing rate of QFT positivity being more pronounced at Zhongmu site than at Danyang site. Consistently, compared to Danyang site, Zhongmu site showed similar QFT reversion rate but much lower QFT conversion rate. These findings suggest that with the decreased TB incidence and new infection rate, the burden of TBI has also decreased significantly with regional differences in rural residents from China.

In our study, we found that although the proportion of the elderly population in China has increased compared with 10 years ago, we still found that the prevalence of TBI among rural residents in 2023 was lower than that reported in 2013.^5^ One possible explanation was that during COVID-19, a number of prevention and control measures were implemented to manage the pandemic, including the use of masks and maintaining effective social distancing, which would result in a lower risk of TB transmission.^13^^,^^14^ Another possible explanation was that the reported incidence of TB has decreased significantly in the past 10 years, resulting in fewer new TBIs. This was supported by the findings of previous studies that a decline in the prevalence of TBI was parallel to the decline in the incidence of active TB.^15^^,^^16^ However, a decrease in the prevalence of TBI due to deaths among older people, who have higher prevalence of TBI, could not be excluded.^5^^,^^17^ Importantly, we found that the declining trend in TB incidence was more pronounced in Zhongmu, along with a lower rate of QFT conversion. We also used the GM (1,1) model to predict the reported TB incidence rate for the next 7 years in the two study sites. It can be seen that with current prevention and control measures, the Zhongmu site could achieve TB incidence of less than 10 per 100,000 by 2030 earlier than the Danyang site, which is the target of the End TB Strategy proposed by WHO. This may be attributed to the fact that more interventions have been carried out in Zhongmu site than in Danyang site over the past 10 years.^18^ However, it cannot be ruled out that the higher proportion of elderly people in Danyang has led to a slower decline in TB incidence and TBI prevalence. First, compared with Danyang site, the Zhongmu site has conducted more than 100,000 person times active TB screening among key populations.^19^^,^^20^ Timely isolation and treatment of active TB patients found during screening could effectively reduce disease transmission. Furthermore, two randomized controlled trial studies were implemented among individuals with TBI in the elderly and in people with inactive TB in Zhongmu site in the past decade,^21^, ^22^, ^23^ and the research results showed that treatment of TBI can effectively reduce the risk of active TB development with an efficacy ranging from 55% to 69%. The interregional variation further supported the idea that regions with significant declines in TB incidence had a lower risk of new infections, and confirmed the importance of active interventions in reducing TB incidence. Therefore, monitoring and evaluation of the TBI burden is important to guide the improvement of TB strategies and should be one of the important elements in the assessment of TB epidemics.

In present study, TST positivity rate has decreased less in the last 10 years as compared to the IGRA positivity rate. This might be related to the specificity of TST, which was susceptible to BCG vaccination and non-tuberculous mycobacteria (NTM) infection.^5^^,^^24^ In 1997, the BCG re-vaccination was cancelled in China, and since then the BCG vaccination has been implemented as a one-time vaccination strategy for newborns. We found that the effect of BCG vaccination on TST results was no longer significant in participants aged 15 years and older. Thus, our results supported that TST could be used for TBI burden assessment in the adult population as well as IGRA without concern for the influence of BCG vaccination on its performance of specificity. In addition, besides BCG vaccination, NTM infection might influence the positivity of TST as well and thus lead to low agreement between IGRA and TST.^5^^,^^25^ It is important to note that BCG vaccination rate in Danyang was low. This might be due to the fact that BCG vaccination was included in the national immunization programme in China since 1978 and those born before 1978 had a lower vaccination rate (24.15%, 703/2911) in Danyang.

For QFT conversion, a reasonable indicator of new infection, we found that it was independently associated with male gender, consistent with our previous findings.^26^ The reason might be that men often have more active social responsibilities than women. For example, in addition to farming, rural men have to go out to work and are therefore at a higher risk of MTB infection exposure. It was not excluding the possibility that women might be more resistant to bacterial infections than males, probably due to sex-biased differences in immune capabilities.^27^^,^^28^ It is worth noting that the participants with history of close contact with TB patients and immunodeficiency had a lower rate of conversion in this study, which was inconsistent with previous finding.^26^ The limited sample size of this study might affect the accuracy of the results. Besides, both two variables were collected through self-reporting, so information bias could not be completely excluded. Thus, this result need to be further verified in the future with larger sample sizes.

In the absence of a gold standard, a stricter definition of QFT reversion has been adopted in this study.^11^ It can be seen that about one third of people in both study sites showed reversion from QFT positive in 2023. In particular, we also observed that about a quarter of participants with baseline QFT+/TST+ results reverted to QFT-/TST-results in 2023. Taken together, these data showed that self-clearance unrelated to anti-TB treatment might be related to changes in the host's autoimmune levels, and clearance of infections due to other health problems and the use of broad-spectrum antibiotics, such as levofloxacin, cannot be ruled out.^29^ In this study, we found that non-smoking was associated with reversion, which was consistent with other studies.^30^^,^^31^ It was possible that smoking could cause immune impairment and changes to the respiratory tract that could increase the risk of persistent MTB infection over time.^32^, ^33^, ^34^ Therefore, it can be seen that smoking cessation campaign is also of great significance for the prevention and control of TB.

There were several limitations to this study. First, our study population was not representative of the registered population in rural China, as many people in the 18–40 age group did not live in their registered households due to the problem of population mobility, such as migration from rural to urban. Moreover, rural men and participants age <50 years were more active in their social work, so the proportion of these population among those who participated in the survey was lower than among those who did not participate. The standardisation of gender and age may also introduce bias due to insufficient population representation. In addition, due to resource limitations, we selected two study sites with relatively stable populations for this investigation among the four sites studied in 2013, which might lead to selection bias. Second, our study found that TB incidence and TBI prevalence have both decreased over the past 10 years, however, the exact correlation between these two indicators cannot be determined as we only have two time points for evaluating TBI prevalence. Third, we used the GM (1,1) model to predict the reported TB incidence rate of the two regions in the following 7 years, without taking into account other factors, so the predicted value may be somewhat different from the actual TB incidence rates. Fourth, we only retested the infection status in 2014 and in 2023 for individuals with negative baseline IGRA results. Changes in TBI among these populations between 2015 and 2022 were unavailable. Finally, potential risk factors such as history of contact with TB patients, smoking, and alcohol consumption were analysed based on participants' reports. Reporting and recall bias cannot be excluded.

In conclusion, our results showed that the prevalence of TBI in rural residents from China has decreased significantly over the past decade. The regional differences in the decline might be explained at least partly by the varied epidemic situation and prevention and control measures implemented in different regions. Our study suggests that dynamic assessment of TBI burden is important for comprehensively understanding the TB epidemic and for more precisely integrating prevention and control strategies.

## Contributors

QJ and LG designed the study. XC, HX, JD, LZ, WD, BF, ZLiu, LW, BZ, DW, JY, WL, CC, GW, FS, HL and WY contributed to data collection. YH, LS, JH, TG, YD, ZLi, JL and YZ were responsible for quality control of the follow-up investigation at the two study sites. XC, LG, JD and QJ did data analysis and wrote the report. XC, HX, LZ, WD, BF, JD, LG and QJ participated in the data interpretation. XC, HX, BF and LG verified the data. All authors had full access to raw data in the study and had the final responsibility for the decision to submit for publication.

## Data sharing statement

The datasets used for this study are available on request from the corresponding author.

## Declaration of interests

The authors declare no competing interests.
