# Supplementary material for: The dynamic change of tuberculosis infection prevalence in rural residents: 10-year follow-up of a population-based, multicentre cohort study from China
Source: Lancet Reg Health West Pac. 2025 Mar 6;56:101509. doi: 10.1016/j.lanwpc.2025.101509 (PMC11930089; doi:10.1016/j.lanwpc.2025.101509)
Supplement: Supplementary Tables [file mmc1.docx]

**The dynamic change of tuberculosis infection prevalence in rural residents: 10-year follow-up of a population-based, multicentre cohort study from China**

Xuefang Cao^1,2*^ PhD, Lei Gao^1,2*^ PhD, Henan Xin^1,2*^ PhD, Limei Zhu^3^ PhD, Weitao Duan^4^ BS, Boxuan Feng^1,2^ BS, Wei Lu^3^ PhD, Zisen Liu^4^ BS, Yijun He^1,2^ MD, Lingyu Shen^1,2^ MD, Juanjuan Huang^1,2^ MD, Bin Zhang^4^ BS, Dakuan Wang^4^ BS, Jiaoxia Yan^4^ BS, Cheng Chen^3^ PhD, Lihui Wang^5^ PhD, Wenhua Yin^5^ BS, Guochen Wang^5^ BS, Tonglei Guo^1,2^ PhD, Yuanzhi Di^1,2^ BS, Zihan Li^1,2^ MD, Jianguo Liang^1,2^ MD, Yaqi Zhao^1,2^ BS, Hongzhi Li^6^ MD, Fei Shen ^6^ MD, Jiang Du^1,2†^ PhD, Qi Jin^1,2†^ PhD

1. NHC Key Laboratory of Systems Biology of Pathogens, National Institute of Pathogen Biology, and Center for Tuberculosis Research, Chinese Academy of Medical Sciences and Peking Union Medical College, Beijing, P.R. China.
2. Key Laboratory of Pathogen Infection Prevention and Control (Ministry of Education), National Institute of Pathogen Biology, Chinese Academy of Medical Sciences & Peking Union Medical College, Beijing, P.R. China.
3. Center for Disease Control and Prevention of Jiangsu Province, Nanjing, P. R. China.
4. Center for Disease Control and Prevention of Zhongmu County, Zhengzhou, P. R. China.
5. Center for Disease Control and Prevention of Danyang City, Zhenjiang, P. R. China.
6. The Sixth People’s Hospital of Zhengzhou, Zhengzhou, P. R. China.

*These authors contributed equally.

†These authors contributed equally.

Correspondence to: Prof. Qi Jin, Institute of Pathogen Biology, Chinese Academy of Medical Sciences & Peking Union Medical College, No 16 Tianrong Street, 102629, Beijing, China, Email: zdsys@vip.sina.com; or Prof. Jiang Du, Institute of Pathogen Biology, Chinese Academy of Medical Sciences & Peking Union Medical College, No 16 Tianrong Street, 102629, Beijing, China, Email:dujiang@ipbcams.ac.cn.

**Table S1. Population sampling included in the analysis of TBI.**

|  | **Danyang site** | **Zhongmu site** | **Total** |
| --- | --- | --- | --- |
| Province | Jiangsu | Henan | NA |
| Geographical location | Eastern China,  plains | Central China,  plains | NA |
| Per capita disposable income in 2023 (RMB) | 53382·1 | 32591·9 | NA |
| Reported incidence of active TB in 2023 (per 100,000 population) | 21·43 | 19·35 | NA |
| Registered population | 8629 (54%) | 7345 (46%) | 15974 |
| Excluded because not defined as resident population | 3287 (64%) | 1834 (36%) | 5121 |
| Excluded because <18 years old | 509(23%) | 1712 (77%) | 2221 |
| Excluded because of pregnancy | 6 (43%) | 8 (57%) | 14 |
| Excluded because of present active pulmonary TB | 0 (0%) | 1 (100%) | 1 |
| Excluded because declined to participate or could not complete study period | 763 (61%) | 490 (39%) | 1253 |
| Eligible population included in the survey | 4064 (55%) | 3300 (45%) | 7364 |
| Excluded because did not participate in the survey despite having  signed consent | 934 (70%) | 399 (30%) | 1333 |
| Excluded because of absent result from IGRA | 3 (8%) | 36 (92%) | 39 |
| Excluded because of history of TB | 30 (47%) | 34 (53%) | 64 |
| Excluded because of present clinically suspected pulmonary TB**†** | 3 (75%) | 1 (25%) | 4 |
| Actual population assessed for the prevalence of TBI | 3094 (52%) | 2830 (48%) | 5924 |

Abbreviation: TB, tuberculosis; IGRA, interferon-γ release assays; TBI, tuberculosis infection.

Data are n (%), unless otherwise indicated. NA=not available.

†If digital chest radiography abnormal and results for IGRA test were positive or TST results were strong positive, we classified cases as having clinically suspected pulmonary TB.

**Table S2. Agreement between the results of the QFT test and the TST test.**

|  | **QFT- /TST -**  **n* (%)** | **QFT+ /TST +**  **n* (%)** | **QFT + / TST -**  **n* (%)** | **QFT - / TST +**  **n* (%)** | **Kappa value**  **(95% CI)** | **Concordant (%)** |
| --- | --- | --- | --- | --- | --- | --- |
| **Total (N=5634)** | 4340 (77%) | 367 (7%) | 394 (7%) | 533 (9%) | 0·35 (0·31, 0·38) | 83·54 |

Abbreviation: QFT, QuantiFERON-TB Gold In-Tube; TST, tuberculin skin test; CI: confidence interval.

*Indeterminate results of the QFT test were excluded.

**Table S3. Univariate and multivariate analysis of QFT positivity.**

| **Variables*** | **QFT positivity** | | |
| --- | --- | --- | --- |
|  | **n/N (%)** | **p for χ² test^§^** | **Adjusted OR**^†^ **(95% CI)** |
| **Age** |  | <0·0001 |  |
| 18-29 years | 7/232 (3%) |  | Reference |
| 30-39 years | 49/490 (10%) |  | 3·49 (1·55-7·86) |
| 40-49 years | 62/563 (11%) |  | 4·14 (1·85-9·23) |
| 50-59 years | 217/1680 (13%) |  | 5·02 (2·32-10·87) |
| 60-69 years | 226/1554 (15%) |  | 5·35 (2·47-11·58) |
| ⩾70 years | 232/1405 (17%) |  | 6·08 (2·80-13·20) |
| **Gender** |  | <0·0001 |  |
| Female | 338/3081 (11%) |  | Reference |
| Male | 455/2843 (16%) |  | 1·25 (0·99-1·58) |
| **Highest education level** |  | 0·526 |  |
| Primary school or lower | 325/2405 (14%) |  |  |
| Middle school | 342/2536 (13%) |  |  |
| High school | 97/705 (14%) |  |  |
| College or higher | 29/278 (10%) |  |  |
| **BMI** |  | 0·324 |  |
| <18·5 kg/m^2^ | 8/87 (9%) |  |  |
| 18·5–<24 kg/m^2^ | 260/2023 (13%) |  |  |
| 24–<28 kg/m^2^ | 347/2441 (14%) |  |  |
| ⩾28 kg/m^2^ | 178/1373 (13%) |  |  |
| **Smoking** |  | <0·0001 |  |
| Never smoker | 455/4018 (11%) |  | Reference |
| Former smoker | 76/482 (16%) |  | 1·22 (0·89-1·66) |
| Current smoker | 262/1424 (18%) |  | 1·64 (1·30-2·09) |
| **Alcohol drinking** |  | 0·008 |  |
| No | 576/4524 (13%) |  | Reference |
| Yes | 217/1400 (16%) |  | 0·87 (0·71-1·06) |
| **Number of BCG scars** |  | <0·0001 |  |
| 0 | 415/2625 (16%) |  | Reference |
| 1 | 206/1799 (11%) |  | 0·84 (0·69-1·01) |
| ≥2 | 162/1463 (11%) |  | 0·69 (0·56-0·84) |
| **History of pulmonary diseases**^#^ |  | 0·223 |  |
| No | 777/5766 (13%) |  |  |
| Yes | 16/158 (10%) |  |  |
| **Self-reported history of close contact with patient with TB** |  | 0·898 |  |
| No | 790/5900 (13%) |  |  |
| Yes | 3/24 (13%) |  |  |
| **Self-reported history of immunological diseases** |  | 0·338 |  |
| No | 786/5851 (13%) |  |  |
| Yes | 7/73 (10%) |  |  |

Abbreviation: QFT, QuantiFERON-TB Gold In-Tube; OR,odds ratio; CI: confidence interval; BMI, body mass index; BCG, bacille Calmette-Guerin; TB, tuberculosis.

*Data might not sum to total because of missing data.

#Including chronic obstructive pulmonary disease, emphysema or chronic bronchitis.

§ A two-tailed p value < 0·20 was considered statistically significant.

†Age and gender were fixed in the multivariable models, other variables with p<0·20 in the univariate model were also entered into the multivariable models.

**Table S4. Declining trend of TB incidence and TBI prevalence detected by QFT in Danyang site and Zhongmu site from 2013 to 2023.**

|  | **Danyang site** | | **Zhongmu site** | |
| --- | --- | --- | --- | --- |
|  | **TB incidence** | **TBI prevalence** | **TB incidence** | **TBI prevalence** |
| The annual rate of decline between 2013 and 2023 | 9·55% | 2·48% | 13·76% | 5·12% |
| Reduction rate from 2013 to 2023 | 63·35% | 22·24% | 77·24% | 40·86% |

Abbreviation: TB, tuberculosis; TBI, tuberculosis infection.

**Table S5. Characteristics of the study participants who participated in 2013 and 2023 examinations.**

| **Variables*** | **N** | **%** |
| --- | --- | --- |
| **Total** | 4648 | 100**%** |
| **Age** |  |  |
| 18-29 years | 170 | 4**%** |
| 30-39 years | 271 | 6**%** |
| 40-49 years | 421 | 9**%** |
| 50-59 years | 1355 | 29**%** |
| 60-69 years | 1259 | 27**%** |
| ⩾70 years | 1172 | 25**%** |
| **Gender** |  |  |
| Female | 2416 | 52**%** |
| Male | 2232 | 48**%** |
| **Highest education level** |  |  |
| Primary school or lower | 1963 | 42**%** |
| Middle school | 1989 | 43**%** |
| High school | 523 | 11**%** |
| College or higher | 173 | 4**%** |
| **BMI** |  |  |
| <18·5 kg/m^2^ | 65 | 1**%** |
| 18·5–<24 kg/m^2^ | 1568 | 34**%** |
| 24–<28 kg/m^2^ | 1929 | 42**%** |
| ⩾28 kg/m^2^ | 1086 | 23**%** |
| **Smoking** |  |  |
| Never smoker | 3106 | 67**%** |
| Former smoker | 437 | 9**%** |
| Current smoker | 1105 | 24**%** |
| **Alcohol drinking** |  |  |
| No | 3573 | 77**%** |
| Yes | 1075 | 23**%** |
| **Number of BCG scars** |  |  |
| 0 | 2183 | 47**%** |
| 1 | 1210 | 26**%** |
| ≥2 | 1243 | 27**%** |
| **History of pulmonary diseases^#^** |  |  |
| No | 4514 | 97**%** |
| Yes | 134 | 3**%** |
| **Self-reported history of close contact with patient with TB** |  |  |
| No | 4626 | 99**%** |
| Yes | 22 | 1**%** |
| **Self-reported history of immunological diseases** |  |  |
| No | 4591 | 99**%** |
| Yes | 57 | 1**%** |
| **QFT test in 2013** |  |  |
| Negative | 3717 | 80**%** |
| Positive | 931 | 20**%** |
| **QFT test in 2023** |  |  |
| Negative | 4036 | 87**%** |
| Positive | 612 | 13**%** |
| **TST induration in 2013** |  |  |
| <5 mm | 2673 | 58**%** |
| 5–9 mm | 782 | 17**%** |
| 10–14 mm | 401 | 9**%** |
| ≥15 mm | 755 | 16**%** |
| **TST induration in 2023** |  |  |
| <5 mm | 3558 | 79**%** |
| 5–9 mm | 191 | 4**%** |
| 10–14 mm | 252 | 6**%** |
| ≥15 mm | 484 | 11**%** |

Abbreviation: BMI, body mass index; BCG, bacille Calmette-Guerin; TB, tuberculosis; QFT, QuantiFERON-TB Gold In-Tube; TST, tuberculin skin test.

*Data might not sum to total because of missing data.

^#^Including chronic obstructive pulmonary disease, emphysema or chronic bronchitis.

**Table S6. Identification of potential factors associated with conversion of QFT by stricter definition among participants with 2013 and 2023 examinations.**

| **Variables*** | **QFT conversion rate^#^, n/N (%)** | **p value**^‡^ | **Adjusted OR**^&^ **(95% CI)** |
| --- | --- | --- | --- |
| **Total** | 46/3375 (1**%**) |  |  |
| **Age** |  | 0·228^†^ |  |
| <60 years | 20/1765 (1**%**) |  | Reference |
| ≥ 60 years | 26/1610 (2**%**) |  | 1·13 (0·58-2·22) |
| **Gender** |  | 0·156^†^ |  |
| Female | 20/1817 (1**%**) |  | Reference |
| Male | 26/1558 (2**%**) |  | 1·61 (0·88-2·94) |
| **Highest education level** |  | 0·147^†^ |  |
| High school or lower | 42/3228 (1**%**) |  | Reference |
| College or higher | 4/147 (3**%**) |  | 3·11 (1·02-9·44) |
| **BMI** |  | 0·354^†^ |  |
| <18·5 kg/m^2^ | 0/49 (0**%**) |  |  |
| 18·5–<24 kg/m^2^ | 19/1128 (2**%**) |  |  |
| 24–<28 kg/m^2^ | 20/1373 (1**%**) |  |  |
| ⩾28 kg/m^2^ | 7/825 (1**%**) |  |  |
| **Smoking** |  | 0·652^†^ |  |
| Never smoker | 30/2317 (1**%**) |  |  |
| Former smoker | 3/284 (1**%**) |  |  |
| Current smoker | 13/774 (2**%**) |  |  |
| **Alcohol drinking** |  | 0·412^†^ |  |
| No | 38/2619 (1**%**) |  |  |
| Yes | 8/756 (1**%**) |  |  |
| **Self-reported history of close contact with patient with TB** |  | 1·000^§^ |  |
| No | 46/3359 (1**%**) |  |  |
| Yes | 0/16 (0**%**) |  |  |
| **Self-reported history of immunological diseases** |  | 0·432^§^ |  |
| No | 45/3334 (1**%**) |  |  |
| Yes | 1/41 (2**%**) |  |  |
| **BCG scar** |  | 0·012^†^ |  |
| Absent | 28/1479 (2**%**) |  | Reference |
| Present | 17/1895 (1**%**) |  | 0·82 (0·38-1·78) |
| **Study site** |  |  |  |
| Danyang | 33/1612 (2**%**) | 0·001^†^ | Reference |
| Zhongmu | 13/1763 (1**%**) |  | 0·34 (0·16-0·76) |

Abbreviation: QFT, QuantiFERON-TB Gold In-Tube; OR,odds ratio; CI: confidence interval; BMI, body mass index; TB, tuberculosis; BCG, bacille Calmette-Guerin.

*Data might not sum to total because of missing data.

#The definition of conversion: the IFN-γ levels of TBAg-Nil from IFN-γ < 0·20 IU/ml in 2013 to > 0·70 IU/ml in 2023.

‡ A two-tailed p value < 0·20 was considered statistically significant.

† p for χ^2^ test.

§ p for Fisher’s exact test.

&The missing cases were removed from the multivariable model. Age and gender were fixed in the multivariable models, other variables with p<0·20 in the univariate model were also entered into the multivariable models.


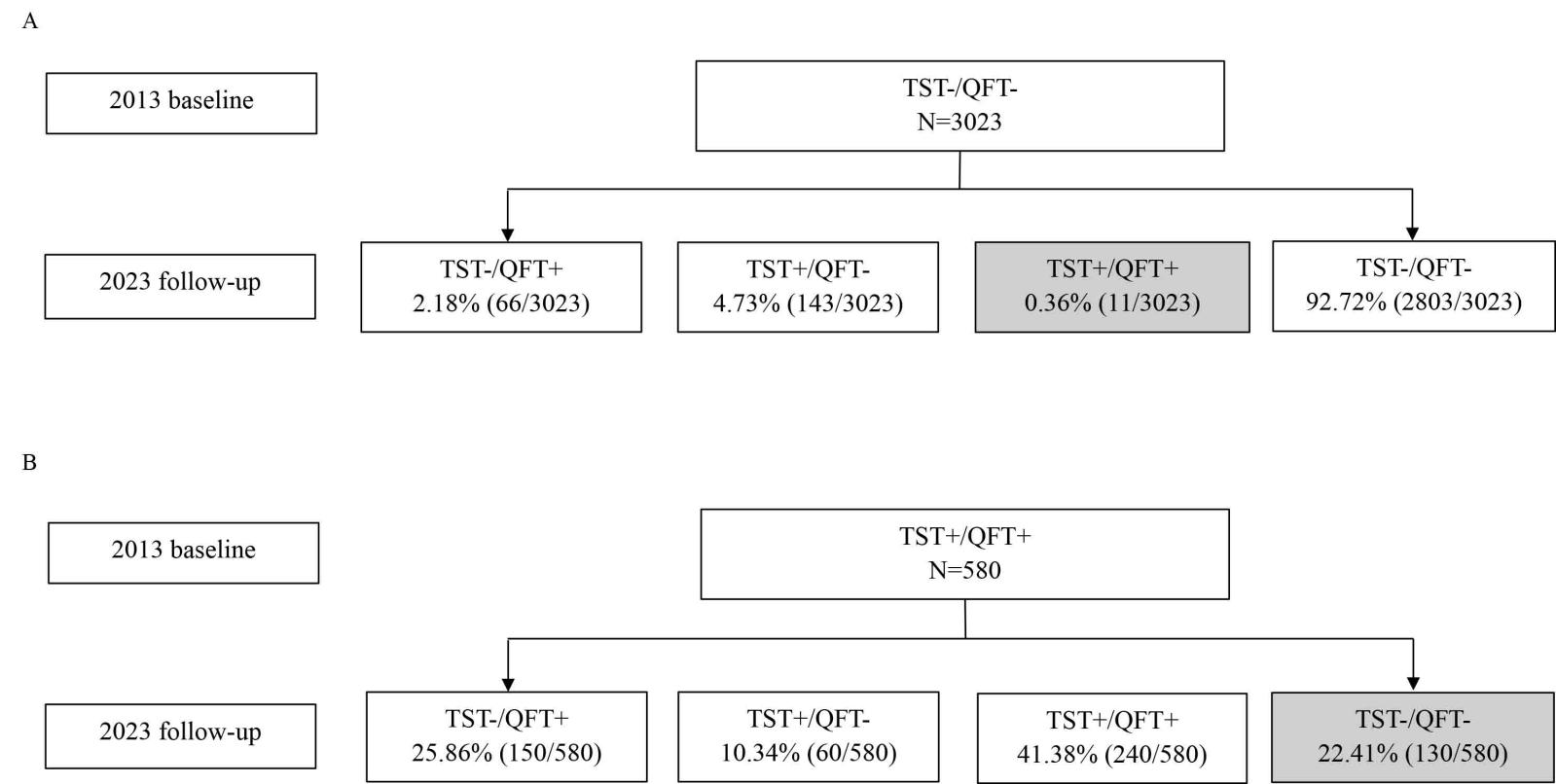


**Figure S1. Change in outcomes at 2023 survey for study participants with baseline QFT-/TST- and QFT+/TST+, respectively.**

1. There were 3023 participants with baseline QFT-/TST- participated in the 2023 survey. Of these, 0·36% (11/3023) showed positive results for both QFT and TST (QFT+/TST+) in 2023.
2. There were 580 participants with baseline QFT+/TST+ participated in the 2023 survey. Of these, 22·41% (130/580) showed negative results for both QFT and TST (QFT-/TST-) in 2023.

**Table S7. Identification of potential factors associated with reversion of QFT by traditional definition among participants with 2013 and 2023 examinations.**

| **Variables*** | **QFT reversion rate^#^, n/N (%)** | **p for χ^2^ test**^§^ | **Adjusted OR**^&^ **(95% CI)** |
| --- | --- | --- | --- |
| **Total** | 431/931 (46**%**) |  |  |
| **Age** |  | 0·002 |  |
| <60 years | 122/312 (39**%**) |  | Reference |
| ≥ 60 years | 309/619 (50**%**) |  | 1·53 (1·16-2·03) |
| **Gender** |  | 0·652 |  |
| Female | 196/416 (47**%**) |  | Reference |
| Male | 235/515 (46**%**) |  | 1·31 (0·90-1·91) |
| **Highest education level** |  | 0·622 |  |
| High school or lower | 425/916 (46**%**) |  |  |
| College or higher | 6/15 (40**%**) |  |  |
| **BMI** |  | 0·450 |  |
| <18·5 kg/m^2^ | 7/10 (70**%**) |  |  |
| 18·5–<24 kg/m^2^ | 149/319 (47**%**) |  |  |
| 24–<28 kg/m^2^ | 188/405 (46**%**) |  |  |
| ⩾28 kg/m^2^ | 87/197 (44**%**) |  |  |
| **Smoking** |  | 0·001 |  |
| Never smoker | 269/552 (49**%**) |  | 2·00 (1·32-3·02) |
| Former smoker | 66/121 (55**%**) |  | 1·97 (1·27-3·07) |
| Current smoker | 96/258 (37**%**) |  | Reference |
| **Alcohol drinking** |  | 0·311 |  |
| No | 323/683 (47**%**) |  |  |
| Yes | 108/248(44**%**) |  |  |
| **History of pulmonary diseases**^‡^ |  | 0·051 |  |
| No | 418/912 (46**%**) |  | Reference |
| Yes | 13/19 (68**%**) |  | 2·21 (0·82-5·95) |
| **Self-reported history of immunological diseases** |  | 0·095 |  |
| No | 422/918 (46**%**) |  | Reference |
| Yes | 9/13 (69**%**) |  | 2·31 (0·69-7·70) |
| **BCG scar** |  | 0·943 |  |
| Absent | 234/503 (47**%**) |  |  |
| Present | 193/417 (46**%**) |  |  |
| **Study site** |  | 0·551 |  |
| Danyang | 242/513 (47**%**) |  |  |
| Zhongmu | 189/418 (45**%**) |  |  |

Abbreviation: QFT, QuantiFERON-TB Gold In-Tube; OR,odds ratio; CI: confidence interval; BMI, body mass index; BCG, bacille Calmette-Guerin.

*Data might not sum to total because of missing data.

‡Including chronic obstructive pulmonary disease, emphysema or chronic bronchitis.

#The definition of reversion: the IFN-γ levels of TBAg-Nil from IFN-γ ≥ 0·35 IU/ml in 2013 to < 0·35 IU/ml in 2023.

§ A two-tailed p value < 0·20 was considered statistically significant.

& The missing cases were removed from the multivariable model. Age and gender were fixed in the multivariable models, other variables with p<0·20 in the univariate model were also entered into the multivariable models.

**Table S8. Comparison of TST positivity rates between with and without BCG vaccination among participants aged 5-14 years in 2013.**

| Participants | **Year** | **TST positivity (≥10 mm), n/N（%）** | | **p for χ² test** |
| --- | --- | --- | --- | --- |
|  |  | **BCG scar** | **No BCG scar** |  |
| 5-14 years in 2013 | 2013 | 17/159 (11**%**) | 2/36 (6**%**) | 0·348 |
|  | 2023 | 35/159 (22**%**) | 7/36 (19**%**) | 0·735 |

Abbreviation: TST, tuberculin skin test; BCG, bacille Calmette-Guerin.

**Table S9. Comparison of TST positivity rates between with and without BCG vaccination among participants aged 5-14 and 15-24 years in 2023.**

| Participants | **TST positivity (≥10 mm), n/N（%）** | | **p for χ² test** |
| --- | --- | --- | --- |
|  | **BCG scar** | **No BCG scar** |  |
| 5-14 years in 2023 | 25/624 (4**%**) | 0/9 (0**%**) | 0·540 |
| 15-24 years in 2023 | 41/227 (18**%**) | 7/38 (18**%**) | 0·958 |

Abbreviation: TST, tuberculin skin test; BCG, bacille Calmette-Guerin.
